# Vascular psychosis in the elderly. case report and literature review for different antypsichotic treatment strategies

**DOI:** 10.1192/j.eurpsy.2021.1973

**Published:** 2021-08-13

**Authors:** T. Gutiérrez Higueras, S. Sainz De La Cuesta Alonso, F. Calera Cortés, S. Vicent Forés

**Affiliations:** Clinical Unit Of Mental Health., Reina Sofia University Hospital., Córdoba., Spain

**Keywords:** Treatment, vascular, psychosis, Elderly

## Abstract

**Introduction:**

The most common cause of vascular psychosis is cerebrovascular disease. Tipically a late-onset in the elderly is accompanied with multiple strokes (disruption of blood flow to the brain). Dementia is a clinical syndrome characterised by cognitive, neuropsychiatric, and functional symptoms. About neuropsychiatric symptoms there are multiple warnings concerning the use of antipsychotics in people with dementia due to an increased risk of death and stroke.

**Objectives:**

Presentation of a case of vascular psychosis with a literature review of antipsychotic drugs used in these cases.

**Methods:**

We carried out a literature review in Pubmed electing those articles focused on antipsychotic treatment options.

**Results:**

A 81-year-old man was taken by his son seeking medical assistance due delusions of reference and auditory hallucinations. He believed that his family wanted to kill him. He had the sensation about how multiple voices were telling him about to scape from people who wanted to kill him. After a completelly study and CT, chronic microvascular infarctions where found. After onset of non effective treatment with haloperidol 1.5 mg during 1 week, we switched into risperidone 1.5mg. Effective treatment was found and now patient is under control of sypmtoms.

**Conclusions:**

Different antypsichotic treatments are described in the literature. Risperidone, quetiapine and olanzapine were found as most used antipsychotic for psychosis in vascular dementia. Comparision of side effect profile of antipsychotic and effectiviness must be the target for an adecuate treatment.

**Disclosure:**

No significant relationships.

